# Distribution and dynamics of electron transport complexes in cyanobacterial thylakoid membranes^☆^

**DOI:** 10.1016/j.bbabio.2015.11.010

**Published:** 2016-03

**Authors:** Lu-Ning Liu

**Affiliations:** Institute of Integrative Biology, University of Liverpool, Crown Street, Liverpool L69 7ZB, United Kingdom

**Keywords:** Cyanobacteria, Electron transport, Membrane protein, Photosynthesis, Protein distribution, Protein dynamics, Respiration, Supramolecular complex, Thylakoid membrane

## Abstract

The cyanobacterial thylakoid membrane represents a system that can carry out both oxygenic photosynthesis and respiration simultaneously. The organization, interactions and mobility of components of these two electron transport pathways are indispensable to the biosynthesis of thylakoid membrane modules and the optimization of bioenergetic electron flow in response to environmental changes. These are of fundamental importance to the metabolic robustness and plasticity of cyanobacteria. This review summarizes our current knowledge about the distribution and dynamics of electron transport components in cyanobacterial thylakoid membranes. Global understanding of the principles that govern the dynamic regulation of electron transport pathways in nature will provide a framework for the design and synthetic engineering of new bioenergetic machinery to improve photosynthesis and biofuel production. This article is part of a Special Issue entitled: Organization and dynamics of bioenergetic systems in bacteria, edited by Conrad Mullineaux.

## Highlights

• Cyanobacterial thylakoid membranes carry out both oxygenic photosynthesis and respiration.
• Electron transport components are located in the thylakoid membrane and functionally coordinate with each other.
• Distribution and dynamics of electron transport components are physiologically regulated in response to environmental change.

## 1 Introduction

Cyanobacteria are the oldest oxygenic phototrophs on Earth. They were not only among the early microbial pioneers of life to produce oxygen that is indispensable for sustaining aerobic life in the atmosphere, but also have consistently served as a predominant contributor to our sustainable environment for around 3.5 billion years [1]. Cyanobacteria can adapt to a variety of environmental changes and have a wide variety of habitats, predominantly ascribed to the robustness and plasticity of their metabolic systems. The thylakoid membranes of many cyanobacteria that have been studied are uniquely capable of conducting both photosynthetic and respiratory electron transductions [2], [3]. The dynamics and modulation of electron transport pathways are essential for cyanobacteria to optimize their metabolism towards environmental challenges. In the last decades, improvements in structural biology techniques have provided substantial information, in molecular detail, about the structures and functions of the electron transport complexes in cyanobacterial thylakoid membranes. However, we still have insufficient knowledge about the overall organization of the thylakoid membranes, the interactions between electron transport complexes and the dynamics of these functional modules. This is a major impediment to addressing many fundamental questions, such as how these electron transport components are biosynthesized and degraded, how their functions are regulated, and how they communicate with each other within the same membrane or between different cellular membranes.

This review focuses on the spatial organization and dynamics of electron transport chains in cyanobacterial thylakoid membranes, and discusses the functional significance of the distribution and mobility of electron transport modules in vivo. The dynamic modulation of the electron flow network produces optimized energy transduction in cyanobacteria. Extensive study of cyanobacterial photosynthetic membranes will provide essential information for the design and engineering of new photosynthetic machinery and devices, with the attempts to improve bioenergy production. In addition, given the close evolutionary relationship between cyanobacteria and chloroplasts, the photoheterotrophic cyanobacteria represent an important model for elucidating the structure and function of chloroplasts in higher plants. Alternatively, knowledge obtained from plant chloroplasts will also inform the study of cyanobacterial thylakoid membranes.

## 2 Cyanobacterial thylakoid membrane structure

### 2.1 Composition

**Fig. 1:**
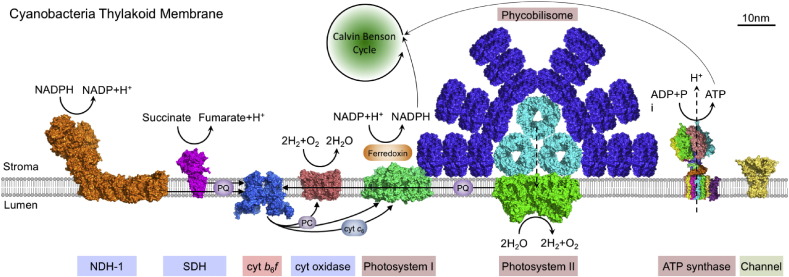
Schematic model of cyanobacterial thylakoid membrane (based on knowledge of *Synechocystis* 6803 thylakoids), showing the interplay of photosynthetic and respiratory electron transport component in the same membrane. Photosynthetic electron transfer complexes include phycobilisome, PSII and PSI, cyt *b*_*6*_*f* and ATPase. The presence of phycobilisome–photosystem supercomplex in vivo has been identified [38], [39]. Complexes specific for respiratory electron transport chain are NDH-1, SDH and cyt oxidase. Some components, such as the cyt *b*_*6*_*f*, PQ and PC are shared by both electron transport pathways. There are also potassium channel proteins in the thylakoid membrane. Arrows indicate the electron transduction reactions. Abbreviations: ADP — adenosine diphosphate, ATP — adenosine triphosphate, cyt *b*_*6*_*f* — cytochrome *b*_*6*_*f*, cyt *c*_6_ — cytochrome *c*_6_, cyt oxidase — cytochrome oxidase, NADP(H) — nicotinamide-adenine dinucleotide phosphate (reduced form), NDH-1 — type 1 NADPH dehydrogenase, PC — plastocyanin, PQ — plastoquinone, SDH — succinate dehydrogenase. The protein structures are achieved from PDB database: allophycocyanin, PDB ID: 1KN1; NDH-1, based on the Complex I structure from *Thermus thermophilus*, PDB ID: 4HEA; cyt *b*_*6*_*f*, PDB ID: 4H13; cyt oxidase, PDB ID: 1OCO; potassium channel protein, based on the *Magnetospirillum magnetotacticum* KirBac3.1 potassium channel crystal structure, PDB ID: 1XL4; phycocyanin, PDB ID: 3O18; PSI, PDB ID: 1JB0; PSII, PDB ID: 3WU2; and SDH, based on the *E. coli* SDH crystal structures, PDB ID: 1NEK.

A unique structural feature of cyanobacterial thylakoid membranes is that it harbors the elements of both photosynthetic and respiratory electron transfer chains, and thereby is capable of performing both oxygenic photosynthesis and aerobic respiration in the same cellular compartment. The major photosynthetic and respiratory electron transport complexes have been structurally characterized in the past decade. Fig. 1 depicts the thylakoid membrane structure based on our current knowledge obtained from a model unicellular cyanobacterium, *Synechocystis* sp. PCC6803 (hereafter *Synechocystis* 6803). The photosynthetic electron transport complexes in the thylakoid membrane include phycobilisome (the membrane associated antenna complex), photosystem II (PSII), photosystem I (PSI), cytochrome (cyt) *b*_*6*_*f* and ATP synthase (ATPase). In addition, there are small electron transport molecules, such as plastoquinone (PQ), plastocyanin (PC) and cytochrome *c*_6_, functioning as electron carriers to shuttle electrons between each electron transport complex and functionally link all the complexes together [4].

Cyanobacteria have evolved the extrinsic supramolecular phycobilisomes associated to the cytoplasmic surfaces of thylakoid membranes, serving as the major antenna for both photosystems [5], [6], [7], [8]. Phycobilisomes are self-assembled supercomplexes composed of chromophore-containing phycobiliproteins and colorless linker polypeptides [9]. The ingeniously-created architecture allows phycobilisomes to absorb efficiently the visible light at the wavelength of 500–670 nm, greatly extending the absorbance range of chlorophyll *a* in photosystems (major absorption at 440 nm and 680 nm). Moreover, stepwise energy transfer within the phycobilisome could also act as a photoprotective mechanism to prevent the photodamage of photosystems by excess light energy [10]. Light energy captured by phycobilisomes is rapidly and efficiently transferred to PSII and PSI. At the reaction center of PSII, a series of light-induced electron transfer reactions occur, leading to the conversion of electrochemical potential energy and water splitting reaction. PQ accepts the electrons from PSII and contributes the electrons to PSI via cyt *b*_*6*_*f* and PC. PSI catalyzes the light-driven electron transport including the oxidation of luminal electron carriers, PC and cyt *c*_6_, and the reduction of ferredoxin. The electron transfer reactions are coupled with the formation of an electrochemical gradient across thylakoid membranes, which is essential for driving ATP synthesis by the ATPase. This electron flow pathway, namely the linear electron transport, is strictly correlated with the evolution of O_2_. During the last steps of electron transfer, the ferredoxin, a strong reductant, transfers electrons to ferredoxin-NADP^+^ oxidoreductase (FNR) to generate NADPH. Apart from the linear electron transport, PSI also participates in the cyclic electron transport, which generates only ATP without any accumulation of NADPH, in order to balance the ratio of ATP and NADPH in the cell [11]. The produced ATP and NADPH will then be utilized for CO_2_ fixation and other cellular metabolism.

Likewise, some protein complexes are also related to the photosynthetic electron flow. A water-soluble orange carotenoid protein (OCP) was shown to mediate directly the fluorescence quenching of phycobilisomes, known as non-photochemical quenching, and possibly in the regulation of energy transfer between phycobilisomes and photosystems [12], [13], [14]. OCP contains a single bound carotenoid (3′-hydroxyechinenone), which can change the conformation between its orange (OCP^O^) and red forms (OCP^R^) [13], [15]. The photoactivated OCP^R^ binds to the phycobilisome core, where it takes excitation energy from phycobilins and converts it to heat as an energy quencher, in order to prevent photodamage of reaction centers at high light. The reversal of OCP-induced energy quenching (conversion of OCP^R^ back to OCP^O^) depends on a second cytoplasmic protein, the fluorescence recovery protein, which binds to OCP and weakens its association with phycobilisomes [16].

In contrast to the well-studied photosynthetic electron transport chain, the respiratory electron transport chain in cyanobacteria is much less understood. The main respiratory electron transport complexes include type-I NAD(P)H dehydrogenase (NDH-1), type-II NAD(P)H dehydrogenase (NDH-2), succinate dehydrogenase (SDH), cytochrome oxidase and alternative oxidases, as well as cyt *b*_*6*_*f* [2]. NDH-1 and SDH were postulated as the principal respiratory electron donor complexes in cyanobacteria [2], [17], [18], [19]. The cyanobacterial NDH-1 complex structurally and functionally resembles mitochondrial Complex I of the respiratory chain, and plays key roles in respiration, cyclic electron flow around PSI and CO_2_ uptake [20]. Electrons from respiratory substrates enter the electron transport chain via PQ reduction by NDH-1 or SDH, and are passed on through cyt *b*_*6*_*f* and the luminal electron carrier, PC or cytochrome *c*. Afterward, they could be transferred to either a terminal oxidase to perform conventional respiratory electron transport with net oxidation of the metabolite pool, or could be transferred to PSI to participate in the cyclic photosynthetic electron transport. The redox state of PQ pool has been demonstrated to play an important role in steering the electron flow into different directions [19].

The components of both photosynthetic and respiratory electron transport chains are structurally and functionally correlated with each other, ensuring highly efficient and optimized electron flow in the cell. Some of these electron transport components, for instance cyt *b*_*6*_*f*, PQ and PC/cyt c, are functionally shared by both photosynthetic and respiratory electron transport pathways [2].

Despite the electron flow components discussed above, there are other components located in cyanobacterial thylakoid membranes. At least five potassium channel homologs have been identified in the genome of *Synechocystis* 6803, based on their sequence similarity to ion channels from other species [21]. A potassium-selective glutamate receptor, which represents an evolutionary link between two transmembrane containing potassium channels and glutamate receptors of eukaryotes, has been characterized [22], [23]. Recent studies have revealed that the SynK, a six transmembrane voltage-sensing potassium channel, is located in both thylakoid and cytoplasmic membranes of *Synechocystis* 6803 [24]. This channel protein is essential to balance the electric component of transthylakoid proton gradient for ATP synthesis, and thereby plays a role in regulating photosynthesis and respiration in cyanobacteria [25]. The primordial cyanobacterium *Gloeobacter* (*G.*) *violaceus* also contains a proton-gated ion channel [26], [27]. The detailed mechanism of ionic channels governing the function and regulation of cyanobacterial photosynthetic membrane remains to be further elucidated.

**Fig. 2:**
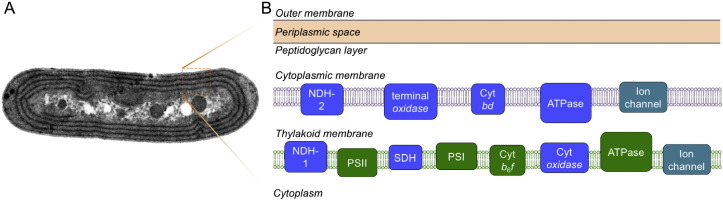
Model of cyanobacterial membrane system and the distribution of electron transport complexes in the membranes. Left, thin-section electron microscopy image of a *Synechococcus* 7942 cell. The thylakoid membranes of *Synechococcus* are organized in a series of regular, concentric layers along the length of the cell. Right, distribution of the major components of cyanobacterial electron transport pathways in the cytoplasmic and thylakoid membranes. Respiratory electron transport components (blue) are located in both cytoplasmic and thylakoid membranes. The thylakoid membrane houses complexes from both photosynthetic (green) and respiratory electron transport chains. Abbreviations: ATPase — ATP synthase, cyt *bd* — cytochrome *bd* oxidase, NDH-1 and -2 — type 1 and II NADPH dehydrogenase, terminal oxidase — cytochrome terminal oxidase.

As shown in Fig. 2, the distribution of photosynthetic and respiratory electron transport chains in cyanobacterial membranes varies depending upon species [28]. Many cyanobacterial species have evolved the intracytoplasmic membrane system, the thylakoid membrane, in addition to the cytoplasmic membrane. In these organisms, the respiratory electron transport chain is located in both cytoplasmic and thylakoid membranes; the thylakoid membrane is endowed with a dual-function of photosynthetic and respiratory electron transport [29]. An exceptional organism is *G. violaceus* that lacks intracytoplasmic membrane in vivo [30], [31]. In *G. violaceus*, the cytoplasmic membrane hosts both photosynthetic and respiratory systems, which intersect and share in part common electron transfer components. Overall, co-existence of respiration and photosynthesis in the same membrane compartment indicates the close functional coordination between the two bioenergetic pathways.

Cyanobacteria are ideal organisms to investigate the processes and structures of electron transport pathways associated with oxygenic photosynthesis and respiration. In particular, two model unicellular cyanobacterial organisms, *Synechocystis* 6803 and *Synechococcus* sp. PCC 7942 (hereafter *Synechococcus* 7942), have been widely used in the laboratory research, because of their completely sequenced genomes and their superior genetic tractability. The spherical-shaped *Synechocystis* 6803 can be grown in several different conditions, with numerous mutants available. The thylakoid membranes in *Synechocystis* 6803 form curved and parallel sheets radiating out from the thylakoid organizing centers. By contrast, the rod-shaped *Synechococcus* 7942 possesses the thylakoid membranes that are organized in a series of regular, concentric cylinders inside the cytoplasmic membrane and surrounding the central cytoplasm. This configuration is advantageous to the study of protein distribution and mobility, especially to one-dimensional fluorescence recovery after photobleaching (FRAP) detection and analysis which is based on live-cell imaging using a confocal fluorescence microscope.

Cyanobacterial thylakoid membranes can not only produce energy, through the electron transport chains, for the cellular metabolism, but also provide the regulatory mechanisms to sense the external stimulation and modulate the cellular metabolism. Current structural model of cyanobacterial thylakoid membranes, based on mainly the atomic structures of individual protein complex and spectroscopic studies, illustrates a static view of the thylakoid membrane architecture. How the complexes and molecules are highly organized in the thylakoid membranes, in a functional context, and how they are dynamically adapted to changing environmental conditions are still poorly understood. Recent advances of analytical techniques, such as cryo-electron tomography, small angle neutron scattering, confocal microscopy and atomic force microscopy [32], have provided new insights into the three-dimensional architecture, dynamics and flexibility of thylakoid membranes.

### 2.2 Membrane heterogeneity

In cyanobacteria, compartmentalization appears to provide a means for metabolic reactions to be concentrated and enhanced within functional protein domains. A typical example is the proteinaceous microcompartment namely the carboxysome, the specialized CO_2_-fixing machinery located in the cytoplasm [33]. The thylakoid membranes also possess compartmentalized modules throughout the membrane network, leading to the formation of spatially separated functional membrane domains and structural heterogeneity of thylakoid membranes, resembling the plant chloroplasts.

The compositional heterogeneity of thylakoid membrane fractions isolated from *Synechocystis* 6803 has been reported [34]. Studies using hyperspectral confocal fluorescence imaging further reported the physical segregation of photosynthetic complexes in *Synechocystis* 6803: the inner thylakoid regions are concentrated with PSI, whereas phycobilisomes and PSII are preferentially located in the outer thylakoids of cyanobacterial cells [35], [36]. Using a combination of electron microscope and immunochemistry, however, it was shown that in *Synechococcus* 7942 the outer thylakoid layer contains mainly ATPase and PSI complexes, whereas PSII and cyt *b*_*6*_*f* are evenly distributed in the outer and inner thylakoid layers [37]. Nevertheless, it is evident that cyanobacterial thylakoid membranes present heterogeneity in protein distribution, indicating the probability that pigment–protein complexes with functional links tend to be in close proximity to and interact with each other.

Indeed, the occurrence of photosynthetic supercomplexes in cyanobacteria has been determined [38], [39]. The megacomplex containing phycobilisome–PSII–PSI was postulated to act as an energy transfer “cluster” that directs excitations to the two photosystems. This megacomplex is less likely to be an electron transport module, as no cyt *b*_*6*_*f* or other electron transport complexes have been identified as parts of the megacomplex [38]. In addition, the physical association of phycobilisome–PSI supercomplex was explored, indicating the potential role of phycobilisomes in the cyclic electron transport around PSI [39]. These studies provided direct evidence for the organizational heterogeneity of electron transport chains in cyanobacterial thylakoid membrane. In green algal chloroplast, the large photosynthetic supercomplex involving PSI-light-harvesting complex I (LHCI), LHCII for PSII, cyt *b*_*6*_*f* and FNR, as well as a membrane-integral complex named PGRL1 was characterized [40]. The assembly/disassembly dynamics of photosynthetic supercomplexes are essential for regulating the energy balance of two photosystems and the pathways of photosynthetic electron flow.

### 2.3 Membrane connection

The biogenesis of cyanobacterial thylakoid membranes is likely to correlate with the development and processing of cytoplasmic membranes [41]. Direct connections between cytoplasmic and thylakoid membranes have been observed in *Synechocystis* 6803 [42], [43], [44]. These connections may provide a media for the communication between cytoplasmic and thylakoid membranes [43]. In addition, the multiple layers of photosynthetic membranes are also connected to each other and form a highly continuous network, reminiscent of the photosynthetic membrane networks found in purple photosynthetic bacteria [45] and higher plant chloroplasts. The long-range membrane network in cells allows the diffusion of water-soluble and lipid-soluble molecules to diffuse continuously through the entire membrane system [44].

### 2.4 Membrane structural flexibility

The structural flexibility of thylakoid membranes is of fundamental importance for the efficiency and regulation of oxygenic photosynthesis at the subcellular scale. Recent studies using small angle neutron scattering revealed that light could induce reversible reorganization of cyanobacterial thylakoid membranes [46], [47]. Variation of the distances between thylakoid layers was considered as a regulatory manner to correlate with many photosynthetic processes in vivo, for instance PSII repair, PC diffusion, protein degradation and non-photochemical quenching, etc. [48]. The size of phycobilisomes was found to play a profound role in determining the spacing between thylakoid membrane pairs [47].

## 3 Distribution and dynamics of photosynthetic complexes in cyanobacterial thylakoid membranes

The formation of functional photosynthetic apparatus is a highly dynamic process, involving de novo protein synthesis, protein self-assembly, turnover and repair, adaptive regulation and crosstalk between components. These orchestrated reactions are crucial for maintaining and optimizing the performance of photosynthetic light-energy conversion. The mobility of protein complexes in thylakoid membranes is governed by a combination of specific protein–protein interactions, membrane viscosity and macromolecular crowding. To date, our knowledge about the organization and dynamics of electron transport components in cyanobacterial thylakoid membranes is still not satisfactory. Some of the pigment–protein complexes in thylakoid membranes are naturally fluorescent, such as phycobilisomes and PSII, allowing direct visualization of the distribution and dynamics of these photosynthetic complexes in vivo without necessity for specific fluorophore binding or fluorescent protein fusion. By contrast, information about the distribution and dynamics of non-fluorescence components is very limited.

### 3.1 Light-harvesting antenna complexes — phycobilisomes

Physical association between phycobilisomes and photosystems is prerequisite for efficient energy flow. The phycobilisome linker protein, ApcE, plays an key role in anchoring phycobilisomes with photosynthetic reaction centers (see review [9]). Hyperspectral confocal fluorescence imaging revealed that phycobilisomes and PSII are predominantly located at the periphery of cyanobacterial thylakoid membranes, whereas PSI is likely distributed in the inner thylakoid regions [35]. This suggested that phycobilisomes have a preferential association with PSII. Furthermore, electron microscopy images revealed the association of phycobilisomes with the stromal (cytoplasmic) surface of PSII rows [49]. Using a chemical cross-linking strategy, a photosynthetic megacomplex composed of phycobilisome, PSII and PSI from *Synechocystis* 6803 has been isolated, indicating the presence of photosynthetic antenna-reaction center supercomplex assembles in cyanobacteria [38]. Time-resolved fluorescence spectroscopy further illustrated that phycobilisomes could channel the excitation energy to the reaction centers of either PSII or PSI, though energy transfer from phycobilisomes to PSI is slower than to PSII [38]. In the cyanobacterium *Anabaena* sp. PCC 7120, a phycobilisome–CpcL–PSI supercomplex was also determined [39]. The CpcL is a linker protein with an N-terminal hydrophilic phycobilisome domain and a C-terminal hydrophobic membrane domain. This specific structure was assumed to be important for combining with both phycobilisome rods and photosystems. Within the supercomplex, PSI is organized into tetramers (in the form of a dimer of dimers) rather than the more general trimers found in vegetative cyanobacterial cells, and the CpcL–phycobilisome rod subcomplexes bind at the periphery of PSI tetramers. It is unclear whether tetrameric PSI is functional in *Anabaena* and whether such specific PSI assembly is only restricted to heterocysts. Likewise, whether the formation of these photosynthetic supercomplexes characterized is transient and dynamic in vivo remains unknown.

Light energy absorbed by phycobilisomes can be physiologically balanced between the two different photosystems in response to changing illumination conditions. This biological process is known as state transitions [50]. Studies using FRAP (see review [51]) revealed that phycobilisomes are mobile and diffuse relatively freely along the cytoplasmic surface of thylakoid membranes [52], indicating the dynamic association and disassociation of phycobilisomes between PSII and PSI. Several factors have been determined to affect the mobility of phycobilisomes and phycobilisome–photosystem association in *Synechococcus* 7942, for instance the size of phycobilisomes, temperature and lipid composition of thylakoid membranes [53]. It was further shown that the phycobilisome mobility is required for state transitions [54] and non-photochemical quenching [55]. Recent study indicated that the phycobilisome mobility also correlates with state transitions in mesophilic red algae [56]. However, in thermophilic red algae, the phycobilisome mobility is greatly restricted, probably due to the strong phycobilisome–photosystem interaction. State transitions in these organisms are replaced by non-photochemical quenching [56].

The rapid diffusion of phycobilisomes has implications for a fluid environment around the thylakoid surface. Such environment will potentially facilitate the large-scale movement of soluble membrane-associated electron transport components such as ferredoxin and flavodiiron (Flv) proteins Flv1–4 [3]. By contrast, FRAP experiments on a cryptophyte alga, in which the light-harvesting antenna are only phycoerythrins instead of entire phycobilisomes and are located uniquely in the thylakoid lumen, illustrated that the diffusion of phycoerythrins is undetectable, implying a particular environment inside the cryptophyte thylakoid lumen [57].

The detailed mechanism governing the rapid phycobilisome diffusion on cyanobacterial thylakoid surfaces still remains to be explored [58]. Atomic force microscopy and electron microscopy images on native thylakoid membranes of the red alga *Porphyridium cruentum* have elucidated significant macromolecular crowding of thylakoid membrane surfaces, with densely packed phycobilisomes [7], [8]. Under such crowd circumstance, the rapid, long-range movement of phycobilisomes is likely to be tremendously restricted by steric hindrance, taking into account the large size of phycobilisomes, the dense lateral packing on thylakoid membrane surface and the limited spacing between opposite thylakoid layers. Given the fact that phycobilisomes and photosystems can form supercomplexes [38], [39] and that PSI can be found in close proximity to PSII [59], it is possible that redistribution of phycobilisomes between the two photosystems could occur only in the local membrane region, rather than the long-range movement [60].

As comparison, LHCs in green alga and plant chloroplasts are very dynamic. They preferentially form supercomplexes with photosystems, such as PSII–LHCII and PSI–LHCI. During state transitions, LHCII can reversibly migrate between PSI and PSII to balance the distribution of absorbed light energy [61]. Under light conditions that preferentially excite PSII (state 2), LHCII is phosphorylated and migrates from PSII to PSI to improve the absorption cross section of PSI–LHCI for harvesting light, whereas the overexcitation of PSI will lead to the dephosphorylation of LHCII and as a consequence, the migration of LHCII back to PSII. The mobility of LHCII has been confirmed by the identification of PSI–LHCI–LHCII supercomplexes from *Chlamydomonas* chloroplasts [62].

### 3.2 Photosystem II

The distribution of cyanobacterial PSII and PSI is relatively homogeneous throughout thylakoid membranes, and often both photosystems are in close proximity [63]. As shown in the hyperspectral confocal fluorescence images, PSII in *Synechocystis* 6803 is organized at the outer thylakoid membranes [35]. In *G. violaceus* that has no thylakoid membranes, PSII complexes are heterogeneously distributed in its plasma membranes, and are preferentially concentrated into specific membrane domains [64]. By contrast, plant thylakoids present spatial segregation of photosynthetic complexes: PSII–LHCII supercomplexes are densely localized in the stacked granal membranes, whereas PSI–LHCI and ATPases are concentrated in the slightly curved stromal lamellae and grana margins [65].

The biosynthesis cycles of PSII complexes are highly dynamic. In cyanobacteria, the assembly of PSII appears to commerce within cytoplasmic membranes and then carries on in thylakoid membranes [41]. This stepwise assembly process relies on the direct membrane connections between cytoplasmic and thylakoid membranes [42], [43], [44]. Different modules assemble together to form a functional PSII monomer, which eventually dimerize and become the final chlorophyll–protein machinery that conduct electron transfer reactions and charge separation [66]. It is evident that stress conditions, such as high light, could lead to a rapid repair cycle for PSII degradation and replacement [67].

It is reasonable that the diffusion of PSII assembly modules in thylakoid membranes plays a role in the dynamic life cycle and regulation of PSII. Compared to the rapid movement of phycobilisomes, however, FRAP studies revealed the membrane-integral PSII complexes are mostly immobile in cyanobacteria under low-intensity white light, probably due to the spatial constrains of macromolecular crowding and specific protein interactions in thylakoid membranes [68]. By contrast, another chlorophyll-binding membrane protein that is supposed to bind with photosystems and response to iron deficiency, the IsiA, was determined to be mobile in thylakoid membranes [68]. It was further revealed that when exposed to intense red light, about 50–60% of PSII changed from a static state to a dynamic state and appear mobile, resulting in a long-range reorganization of PSII in thylakoid membranes [69]. The red-light-induced mobility and structural rearrangement were deduced to correlate with the PSII repair cycle. In addition, lipids in thylakoid membranes were proved to be mobile using fluorescently staining dye [70]. These findings have explicit implications for the mobile configuration of cyanobacterial thylakoid membranes, which is instrumental in protein turnover and repair. Whether the PSII mobility in cyanobacteria is affected by light intensity awaits experimental verification.

The variation of PSII mobility under distinct environmental conditions indicated the existence of specific protein–protein interactions or protein–lipid interactions that govern the functional organization of PSII in cyanobacterial thylakoid membranes. Cyanobacterial thylakoid membranes have exceptionally high protein abundance, which is an impediment to rapid protein mobility [63]. On the other hand, high protein density is advantageous to concentrating chromophores and electron transport complexes into a limited membrane region, and thereby enhancing the efficiency of light capture and photosynthetic performance. The dense packing of protein complexes has also been seen in the photosynthetic membranes of purple bacteria and higher plants [32], [71], [72], [73], [74]. FRAP experiments on higher plant thylakoid membranes reported that 75% of fluorophores are immobile, probably due to the dense packing of protein complexes in grana membranes. However, the rest 25% fraction diffuses freely and rapidly [75], distinct from the immobility of cyanobacterial PSII. The mobility of chlorophyll–protein complexes observed could be ascribed to the movement of LHCII between the grana and stromal lamellae for energy redistribution in state transitions, or the movement of PSII which could be essential for the PSII repair cycle [75]. In addition, the mobility also presents variation in the grana and stromal lamellae. About 50% of chlorophyll–protein complexes are mobile in the unstacked thylakoids, whereas only 20% are mobile in the stacked grana membranes [76]. The discrepancy in protein lateral diffusion very likely reflects the confined environment led by the stacking of grana layers and the high protein/lipid ratio presented in grana.

### 3.3 Photosystem I, cytochrome b_6_f, ATP synthase and ferredoxin-NADP^+^ oxidoreductase

The stoichiometry of PSI and PSII in thylakoid membranes is dynamically regulated in response to light quality and quantity. In *Synechocystis* 6803, the ratio of PSI monomers to PSII monomers varies between 1.5:1 under light favoring PSI and 3:1 under light favoring PSII [77]. In addition, the ratio of PSI to PSII is greater under low-intensity white light than that under high-intensity white light [78].

Though PSI is naturally fluorescent, most of the room-temperature chlorophyll fluorescence is attributed to PSII rather than PSI. This has led to very limited information about the localization and dynamics of PSI in cyanobacterial thylakoids, relative to the extensive studies on PSII. Similarly, despite the profound roles of cyt *b*_*6*_*f*, ATPase and FNR in cyanobacterial electron flow, so far there is no experimental data on the distribution and mobility of these non-florescence complexes. Our limited knowledge about the organization and dynamics of PSI, cyt *b*_*6*_*f* and ATPase is achieved mainly from the studies of bacterial membranes [79], [80] and plant chloroplasts [76].

In higher plants, PSI–LHCI and ATPases are preferentially concentrated in unstacked thylakoid regions, physically separated from PSII–LHCII supercomplexes in the stacked granal membranes. Cyt *b*_*6*_*f* complexes are more evenly distributed throughout chloroplast thylakoid membranes [76]. It is postulated that the lateral mobility of PSI–LHCI in plants might be higher, as they are distributed in the unstacked thylakoid membranes where protein complexes are less densely packed [76]. In addition to fluorescence imaging, an alternative approach, in situ electrophoresis of membrane components within the membranes, was exploited previously to measure the electro-photoluminescence that originates from PSI only and determine the electrophoretic mobility of PSI in chloroplast thylakoid membranes [81]. It is worthy noting that the prepulse field strength used in this technique may cause aggregation of charged particles, and thereby affect the measurement accuracy. The in vivo dynamics of FNR have been suggested by the fact that FNR can interact with several major photosynthetic complexes including cyt *b*_*6*_*f* [82], PSI [83] and NDH-1 [84], [85]. Recently, FNR has been detected in a photosynthetic supercomplex composed of PSI, LHCI, LHCII and cyt *b*_*6*_*f* in plants [40]. By contrast, cyanobacterial FNR contains a phycobilisome linker protein and thus associates with the phycobilisome [86]. No association of cyanobacterial FNR with cyt *b*_*6*_*f* was detected [82]. Future microscopic imaging on fluorescently labeled PSI, cyt *b*_*6*_*f* and ATPase will advance our understanding of the in vivo dynamics and functionality of these electron transport complexes in cyanobacterial photosynthetic membranes.

## 4 Distribution and dynamics of respiratory complexes in cyanobacterial thylakoid membrane

In cyanobacterial thylakoid membranes, the respiratory complexes are laterally interwoven and have functional correlation with the photosynthetic complexes. The exact location of cyanobacterial NDH-1 complexes is controversial. Using different biochemical methods, NDH-1 complexes have been found in both cytoplasmic and thylakoid membranes of *Synechocystis* 6803 [87], explicitly in cytoplasmic membranes of *Anabaena* sp. PCC 7120 [88], or only in thylakoid membranes of *Synechocystis* 6803 [89], [90], [91]. To avoid the variations in the purity of membrane preparations achieved from different biochemical isolation approaches, a recent study used a combination of fluorescence labelling and confocal fluorescence imaging, and detected NDH-1 and SDH of *Synechococcus* 7942 are both confined exclusively in thylakoid membranes. Likewise, cyt oxidase was also found mostly in thylakoid membranes of *Synechocystis* 6803 [92], [93].

**Fig. 3:**
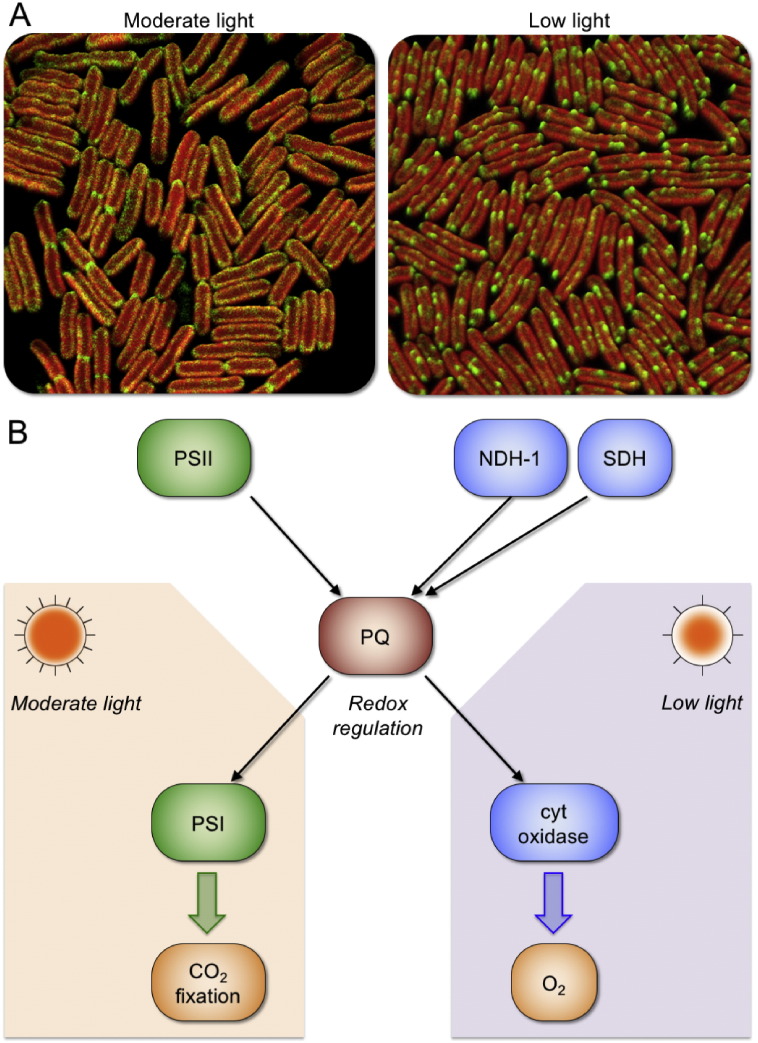
Regulation of electron transport pathways. A, Confocal microscopy images show different localization of functional NDH-1 complexes in *Synechococcus* 7942 grown in moderate light (60 μE m^− 2^ s^− 1^) and low light (6 μE m^− 2^ s^− 1^). The fluorescence of green fluorescence proteins (shown in green) revealed the distribution of NDH-1 complexes, whereas chlorophyll fluorescence (shown in red) indicates the location of thylakoid membranes. The reorganization of NDH-1 complexes in thylakoid membranes highly correlates with the direction of electron flow to respiratory pathway or photosynthetic pathway. For details, see [19]. B, Model of the regulation of electron transport pathways in cyanobacterial thylakoid membranes. Arrows indicate the directions of electron flow. Complexes related to photosynthesis are shown in green and respiratory components are indicated in blue. Different light intensities could trigger the variation of the redox state of PQ pool, and thereby lead to distinct pathways of electron transport.

Our knowledge about the distribution and dynamics of respiratory electron transport complexes in cyanobacterial thylakoid membranes is fragmentary. The study of the distribution of NDH-1 and SDH complexes in *Synechococcus* 7942 provided new insights into the large-scale reorganization and coordination of photosynthetic and respiratory electron transport pathways in cyanobacterial thylakoid membranes [19]. In low-light adapted cells, both these complexes are clustered in discrete membrane zones, suggesting that there is likely spatial segregation of respiratory and photosynthetic complexes in thylakoid membranes. After exposure of these cells to moderate light, a long-range redistribution of both complexes takes place, and these respiratory complexes appear rather evenly distributed in the membrane (Fig. 3) [19]. By this way, respiratory complexes are laterally adjacent to photosynthetic complexes. These results indicated specific interactions between different electron transport chains, which can be triggered by light intensity. It was further demonstrated that the large-scale distribution of respiratory complexes is of functional significance. Moderate light exposure results in a major change of the probability that electrons from the respiratory complexes are transferred to a PSI rather than to a terminal oxidase. Moreover, experiments in the presence of electron transport inhibitors, 3-(3,4-dichlorophenyl)-1,1-dimethylurea (DCMU) and 2,5-dibromo-3-methyl-6-isopropyl-benzoquinone (DBMIB), demonstrated that the reorganization of respiratory complexes in thylakoid membranes is controlled in response to a redox switch of PQ pool that is triggered by light. Oxidation of PQ pool induces the clustering of NDH-1 in segregated thylakoid membrane zones, whereas reduction of PQ pool induces a post-translational switch in the distribution of respiratory complexes through closer association of NDH-1 and SDH with PSI. This biological “switch” provides a basis for regulating the prevalence of linear and cyclic electron flow. In addition, the membrane reorganization (and associated changes in electron transport pathways) occurs on a timescale of about 30–60 min. During this period the spots of NDH-1 remain stationary, but gradually lose intensity as complexes diffuse out of the NDH-1 clusters and spread into the bulk thylakoid membranes [19]. The slow kinetics of the transition is rationalized by giant NDH-1 complexes and highly confined mobility of membrane-integral complexes in cyanobacterial thylakoid membranes. However, specific protein organization and modulation in thylakoid membranes might be adopted to facilitate the rearrangement of large electron transport complexes. These observations indicated that the organization of electron transport complexes in the membrane, at the sub-micron scale, is under physiological control, and plays a crucial role in controlling pathways of electron flux.

Another example of dynamic “electron valves” in cyanobacteria is the Flv proteins, Flv1–4. They are cytoplasmic proteins that take electrons from the photosynthetic electron transport chain and divert them to alternative acceptors. Flv1 and Flv3 form a heterodimer that takes electrons from the acceptor side of PSI and uses them to reduce oxygen [94]. An Flv2/Flv4 heterodimer takes electrons from the acceptor side of PSII, passing them to an unknown acceptor [95]. The regulatory mechanism of the activities of Flv proteins is still unknown.

## 5 Dynamics of electron carriers in the cell membrane and thylakoid lumen

**Fig. 4:**
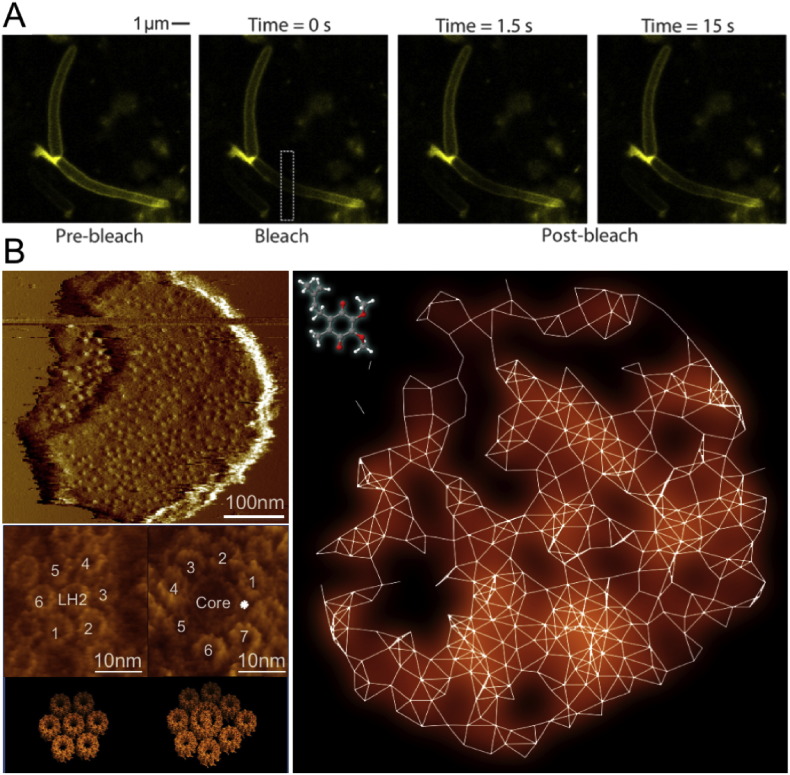
Mobility and diffusion pathways of electron carriers in the cellular membrane. A, Fluorescence recovery after photobleaching of living *E. coli* cells incubated with NBDHA-Q indicated the ubiquinone mobility in bacterial membrane. For details, see [79]. B, Network of quinone pathways within the entire intracytoplasmic photosynthetic membranes from purple photosynthetic bacteria. Overview atomic force microscopy images and molecular-resolution images of typical photosynthetic protein assemblies of the native photosynthetic membranes from *Rhodospirillum photometricum* revealed specific protein organization and crowding in photosynthetic membranes, which are the fundamental basis of the long-range quinone pathways in cellular membranes. For details, see [73].

In addition to the mobility of protein complexes, the diffusion dynamics of small electron transport molecules, comprising PQ, PC and cyt c, are also essential to electron transport [79]. PQ diffuse through the lipid layer of thylakoid membranes, due to their hydrophobic features, and shuttle electrons between PSII and cyt *b*_*6*_*f*. The redox state of PQ pool has been found to function as the key controller of a number of biological reactions in cyanobacteria, including the distribution of respiratory complexes [19], photosystem composition [96], and state transitions [97] and the modulation of circadian clock [98], [99]. The lipid composition and mobility are important factors in determining the mobility of PQ. A recent study explored the in vivo distribution and dynamics of the electron carrier ubiquinone in bacterial bioenergetic membranes, using a green fluorescent ubiquinone analogue named NBDHA-Q [79]. The *Escherichia* (*E.*) *coli* cell suspension was pre-incubated with NBDHA-Q prior to confocal imaging. This allowed the incorporation of NBDHA-Q into cell membranes, in specific the cytoplasmic membrane. There seems no PQ microdomain in the thylakoid membrane, as confocal imaging revealed relatively homogeneous halos along the cellular membrane, without any significant spotty distribution [79]. By contrast, respiratory complexes are concentrated in separate mobile domains in the membrane. Distinct from the respiratory complexes characterized in mitochondria, bacterial respiratory complexes do not present significant co-localization and thereby no supercomplex assembly [79]. FRAP experiments illustrated a rapid diffusion of NBDHA-Q molecules, at the micrometer scale, in *E. coli* cytoplasmic membranes (Fig. 4). The diffusion of NBDHA-Q was further demonstrated to be temperature-dependent and highly correlated with the respiratory activity of *E. coli* cells grown at different temperatures [79]. These results indicated that the electron carriers are highly mobile in the cell membrane, allowing electron transfer between electron transport functional domains.

Another electron carrier, PC, diffuses in the lumen of the thylakoid membrane to connect cyt *b*_*6*_*f* and PSI. Therefore, its mobility is largely affected by the circumstance of thylakoid lumen. It was revealed that light intensity could induce variations of the thylakoid lumenal space [46], [47]. The swelling of thylakoid lumen could then lead to changes in protein dynamics. Indeed, the protein diffusion in the cryptophyte thylakoid lumen is highly restricted, probably due to the crowding environment inside the cryptophyte thylakoid lumen [57]. Further characterization of the environmental conditions of cyanobacterial thylakoid lumen is critical for better understanding the mobility of the lumenal electron carriers.

The organization of photosynthetic membranes plays an important role in regulating the direction and efficiency of electron flow. A common feature of all photosynthetic membranes that have been analyzed is the exceptional protein crowding [32]. As discussed earlier, the dense packing of multicomponent photosynthetic complexes is favorable for excitation energy transfer between complexes on one hand, but on the other hand it significantly reduces the lipid content and space between protein complexes. As a consequence, the membrane fluidity that is required for the diffusion of hydrophobic electron/proton transport carriers (i.e. quinone molecules) is remarkably restricted [74]. It represents an obstacle for efficient electron transport between electron transport complexes in the membranes. Analysis of the architecture of bacterial photosynthetic apparatus proposed a continuous ‘lipid area network’ for the long-range quinone diffusion throughout the photosynthetic membranes of purple bacteria (Fig. 4). The specific quinone pathways are created in nature by the combination of specific local molecular environment and long-range protein organization [73].

## 6 Conclusions

Evolution has developed strategies to modify, in an efficient manner, the electron transport pathways in response to diverse environments. Dynamic organization of electron transport components in cyanobacterial thylakoid membranes provides a basis for the adaptive responses that allow the regulation and optimization of both photosynthesis and respiration. Our current understanding of the functional and structural dynamics of electron transport pathways needs to be substantially improved. For example, further characterization of the dynamics of cyanobacterial PSI, cyt *b*_*6*_*f*, ATPase, cyt oxidase as well as the electron carriers of photosynthetic and respiratory electron transport are imperative. In addition, little is known about the factors that govern the dynamic processes of electron transport pathways in thylakoid membranes, such as how do the lipid composition and lipid–protein interaction affect the organization and dynamics of proteins and molecules involved in electron transport. Global picture of the regulation and acclimation of the electron transport pathway network in cyanobacterial thylakoid membranes will benefit from the recent advances of “omics” approaches, including transcriptomics, proteomics and metabolomics.

Advanced knowledge of the electron transport in photosynthetic membranes has led to attempts to modulate the photosynthetic building blocks and pathways, using the powerful tools of synthetic engineering. In this context, cyanobacteria can be used as an attractive “green” basis for producing high-value compounds, such as food, pharmaceuticals, and biofuels. Understanding the regulatory mechanisms underlying the larger-scale distribution and dynamics of electron transport modules will provide essential information required for developing strategies to optimize and control electron transport pathways, and bioengineering of new bioenergetic machinery or organisms to boost biofuel production.

## Transparency document

Transparency document.
